# From DNA damage to systemic immunity: Interaction networks in radio-immunotherapy

**DOI:** 10.1016/j.cellin.2026.100360

**Published:** 2026-09-12

**Authors:** Ruixue Huang

**Affiliations:** Xiangya School of Public Health, Central South University, Changsha, Hunan, 410078, China

**Keywords:** Cancer, Radiotherapy, Immunotherapy, Molecular interactions, Protein–protein interaction

## Abstract

Immune checkpoint inhibitors (ICIs) have transformed cancer treatment, yet primary and acquired resistance continues to limit durable benefit. Radiotherapy, long valued for its direct cytotoxicity, is now recognized as a powerful immunomodulator that remodels the tumor microenvironment (TME) and can convert immunologically “cold” tumors into “hot” ones. However, ionizing radiation exerts both immune-activating and immunosuppressive effects that vary with dose, fractionation, and biological context, and the therapeutic synergy of radiotherapy and immunotherapy ultimately depends on the molecular interactions that govern these responses. In this review, we first examine how radiation shapes the inflammatory response and both innate and adaptive immunity, including the immunostimulatory potential of low-dose radiation on cellular and humoral immunity, the abscopal and RadScopal effects, cGAS–STING-mediated nucleic-acid sensing, and immunogenic cell death. We then propose molecular interactions as a unifying framework for dissecting therapeutic response and resistance, systematically analyzing protein–protein, protein–DNA, gene–drug, and protein–drug interactions that regulate key signaling pathways and define actionable therapeutic targets. Building on this framework, we summarize current targeting strategies including cytokine therapy, antibody-based immunotherapy and immune checkpoint blockade, and tumor vaccines and review clinical evidence on combined radio-immunotherapy across tumor types. Finally, we discuss critical challenges and future directions, including the optimal sequencing and scheduling of the two modalities, the choice of radiation dose and particle type, the “badscopal” effect, biomarker-guided patient selection, and nanotechnology-enabled delivery. We argue that a systems-level shift from a “local” to a “systemic” view of radiotherapy, grounded in a deeper mastery of these molecular interaction networks, is essential for designing the next generation of precision radio-immunotherapy.

## 1 Introduction

### 1.1 Cancer prevalence and disease burden in the world

According to GLOBOCAN 2022 estimates, lung cancer was the most commonly diagnosed cancer worldwide, while female breast cancer ranked second, with approximately 2.30 million estimated new cases in 2022 (Filho et al., 2025). As a major global health burden, cancer has become the leading cause of death in most countries and regions (Lin et al., 2021). In 2019, more than 10 million people died from cancer globally, approximately twice the number in 1990 (Lin et al., 2021). Global cancer burden has continued to increase in terms of mortality and morbidity, although trends vary substantially by cancer type and region. For instance, approximately 1.8 million lung-cancer-related deaths reportedly occurred in 2022, with an age-standardized incidence rate (ASIR) and age-standardized mortality rate (ASMR) of 23.6 per 100 000 and 16.8 per 100 000, respectively (Guo et al., 2024). Given its significance as a major global health concern, understanding the molecular mechanisms underlying diverse responses to cancer therapies is crucial for advancing treatment strategies.

### 1.2 The challenges of the diverse effects of radiotherapy on immunity

Radiotherapy can be delivered with curative intent in selected localized or locally advanced cancers, either alone or as part of multimodality treatment, and is also used for local control or palliation in metastatic disease. Evidence implies that radiation treatment induces cytotoxicity by triggering DNA damage, especially DNA double-strand break (DSB) in cancer cells, leading to cell death through interphase apoptosis or mitotic catastrophe (Harary et al., 2024). Although radiotherapy has improved treatment outcomes and advanced cancer management, it still faces several challenges. Radiotherapy resistance is a major obstacle in improving treatment efficacy and enhancing the prognosis of cancer (Lin et al., 2024). Cancer cells may develop multiple strategies to counteract radiation damage. As is known, after ionizing radiation, cancer cells may initiate DNA damage repair-related signaling pathways, in which NHEJ (non-homologous end joining) could be a more important DSB repair process than that of HR (homologous recombination) to rapidly rejoin the DSBs (Tan et al., 2023). Radiation can also activate other intrinsic anti-death molecules and signals to build cancer cells with acquired radioresistance. For example, Yuan et al. reported that microRNA-410 may enable non-small-cell lung cancer to acquire radioresistance through targeting the PTEN/PI3K axis (Yuan et al., 2020). Fan et al. indicated that protein–protein interactions (PPIs) between ZNF131 and BACH1 following radiation exposure enhance RAD51-dependent homologous recombination repair, leading to radiotherapy resistance (Fan et al., 2024). Additionally, normal tissue damage such as radiation-induced lung fibrosis is also a larger obstacle to restrain the radiotherapy efficacy. Ionizing radiation can reshape innate and adaptive immune responses, producing both immunostimulatory and immunosuppressive effects depending on dose, timing, tissue context and concomitant therapy (Dai et al., 2025; Wang, Lynch, et al., 2024). Ionizing radiation has significant effects on the tumor immune microenvironment (Zhu et al., 2022). All of these effects contribute to the alteration of the radiotherapy response. On the other hand, ionizing radiation can also trigger the anticancer immunity and immune reprogramming (Cao et al., 2023). Therefore, understanding how and why cancer cell immune responses are influenced by radiation may provide crucial insights into overcoming radiotherapy resistance. This knowledge could aid in the development of more effective and precise immunotherapy strategies for cancer treatment.

## 2 Broad-spectrum mechanisms of radiation-induced immune responses

### 2.1 Inflammation as the common substrate of the radiation response

The immune inflammatory response represents a fundamental biological process that plays a dual role in cancer pathogenesis and therapy. In the context of tumor biology, inflammation serves as a critical component of the tumor microenvironment, actively shaping cancer progression from initiation to metastasis (Wang et al., 2025). Chronic inflammation can promote oncogenesis by generating DNA-damaging free radicals, activating pro-survival signaling pathways, and creating an immunosuppressive milieu that facilitates immune evasion (Xie et al., 2025). Conversely, acute inflammatory responses can mediate antitumor effects through the activation of innate immune cells such as natural killer cells and macrophages, along with the recruitment of adaptive immune components including cytotoxic T lymphocytes (Liu et al., 2024). The complex interplay between neoplastic cells and inflammatory mediators establishes a dynamic ecosystem where cytokines, chemokines, and growth factors collectively influence tumor behavior and therapeutic responses. Understanding this intricate balance is particularly relevant for modern cancer immunotherapy, where strategies such as immune checkpoint inhibition fundamentally rely on modulating inflammatory pathways to enhance antitumor immunity.

Radiation enters this inflammatory ecosystem as a controlled and quantifiable perturbation. Many radiation-induced immune consequences arise from inflammatory and damage-sensing events, whose magnitude and duration are strongly influenced by total dose, dose per fraction, irradiated volume and tissue vascularity, as well as tumor-intrinsic biology, baseline immune contexture and treatment sequencing.

### 2.2 Dose dependence of the radiation-immune response: low-dose radiation as an illustrative case

Definitions of low-dose radiation are context dependent. In the radiation-protection literature, low-dose exposure is generally defined as an absorbed dose below 0.1 Gy, whereas in tumor-directed radiotherapy the term low dose refers to single fractions of approximately 0.5–2 Gy delivered to non-primary lesions. Reported immunomodulatory effects are model-, dose-, fractionation- and tissue-dependent, and these two usages are not interchangeable; throughout this review, low-dose radiation is used in the tumor-directed sense unless stated otherwise (Yang et al., 2016).

The term RadScopal effect is often applied loosely to any immunomodulatory action of low-dose radiation. We use it here only in the narrower sense intended by its originators, that is, as the name of a specific triple-combination strategy, and we describe the general immunomodulatory actions of low-dose radiation without that label. A number of studies have reported immunomodulatory effects of low-dose radiation. The concept of the RadScopal effect was coined by Welsh and colleagues to describe a strategy that combines low-dose and high-dose radiotherapy to different lesions (Barsoumian et al., 2020). It is a combining triple therapy approach (high-dose radiotherapy + low-dose radiotherapy + immunotherapy), in which high-dose radiotherapy directs at primary tumors, low-dose radiotherapy deliver to secondary tumors. Through their investigation, they combined high-dose radiation to “prime” T cells at the primary cancer site, with low-dose radiation targeted to metastatic cancers to “modulate the stroma”. This strategy showed the low-dose radiation has benefits to reprogram the cancer environment and improve the infiltration and function of effector immune cells into secondary cancers (Barsoumian et al., 2020). Zhou et al. conducted a prospective phase I study. Patients received stereotactic body radiotherapy of 30 Gy for small lesions and low-dose radiation of 2 Gy for large lesion concurrently followed by PD-1 inhibitor. The regimen was feasible and yielded a median progression-free survival of 8.6 months; efficacy and any association with PD-1/PD-L1 status require validation in controlled trials using prespecified assays and thresholds (Zhou et al., 2023). The RadScopal rationale is that high-dose irradiation of selected lesions may provide antigenic priming, whereas low-dose irradiation of other lesions may, in some settings, remodel suppressive stroma and improve immune-cell trafficking; both components can have immunostimulatory or suppressive effects depending on context. Thus, low-dose radiation serves as a potential tool to improve the immunogenicity of radiotherapy, and it has also been reported to remodel the tumor immune microenvironment and enhance the effect of anti-CTLA-4 therapy (Dong et al., 2025). Further research is needed to understand fully its therapeutic potential.

The immunomodulatory actions of low-dose radiation on macrophages, vasculature and lymphocyte trafficking are properties of the dose. The RadScopal effect is a treatment design that exploits those properties by combining a high dose to a primary lesion with a low dose to secondary lesions and with systemic immunotherapy, and evidence for the strategy should not be read as evidence for the dose alone, or the reverse.

### 2.3 Radiation and systemic immunity: humoral and lymphocyte-subset evidence

Humoral immunity mainly produces antibodies via B lymphocytes to combat extracellular pathogens and the toxins produced. Because these antibodies are present in bodily fluids, including blood, lymphatic fluid and tissue fluid, this arm of immunity is termed humoral immunity. The major cellular and molecular components of humoral immunity include B cells and plasma cells that produce immunoglobulins, with helper T cells and antigen-presenting cells such as dendritic cells (DCs) and macrophages providing antigen presentation and regulatory signals. The function of humoral immunity defend against extracellular threats such as pathogens and toxins, generating immune memory to achieve long-term immunity through B cells, and providing systemic protection by antibodies’ circulation throughout the body with the blood. The study of effects of low-dose radiation on humoral immunity has various results. The results of low-dose radiation role in early study on occupational workers were inhibition function whereas recent reports are stimulating effects. An early study among residents of territories contaminated with radionuclides after Chernobyl accident showed low-dose radiation exposure led to a decrease in the counts of large granular lymphocytes (Asfandiiarova et al., 1998). Another study showed a 0.1 Gy dose of radiation had an immunosuppressing effect through the inactivation of helper cells in the early period of a postnatal ontogeny (Sharetskii & Abramova, 2006). Bogdándi et al. found that low doses altered splenocyte subset responses and decreased Th1- and Th2-type cytokine expression, whereas B cells were relatively resistant at low doses but became among the most sensitive subsets after 2 Gy exposure (Bogdándi et al., 2010). Gyuleva et al. assessed humoral immune parameters among nuclear power plant workers with the low-dose radiation exposure within the range of 0.06 to 766.36 mSv and found while less than 100 mSv low prevalence of Th1 immune response but Th2 response occurred at doses beyond 100 mSv (Gyuleva et al., 2015). A study among interventional cardiologists showed a significant increase in the expression of the activation marker CD69 on stimulated CD4^+^ T cells, together with altered serum immunoglobulin G levels (Zakeri et al., 2010). Schoenfeld et al. performed a phase II trial in patients with metastatic NSCLC, in that patients received durvalumab (anti-PD-L1) plus tremelimumab (anti-CTLA-4) immunotherapy alone or with low-dose (0.5 Gy) or hypofractionated radiotherapy (24 Gy). They found no difference within immunoradiotherapy alone and low-dose radiation group and no survival improvement. They speculated that lymphocyte death may have blunted the favorable immunological function of radiotherapy (Schoenfeld et al., 2022). Taken together, these humoral data are less consistent than the cellular data, and they are best interpreted as evidence that B cell and antibody compartments are sensitive to dose, to species and to the timing of sampling, rather than as evidence of a uniform stimulatory effect.

### 2.4 Ionizing radiation damage and the innate immune response

Innate immunity is the first line of defense against invading pathogens and cellular damage (Valentini et al., 2021). In general, innate immunity comprises myeloid cells/macrophages, natural killer (NK) cells, and DCs (McMahon et al., 2023). Radiation can stimulate, affect, and regulate the innate immune response. The relationship between radiation and innate immunity can be summarized into certain key aspects:

(1) Radiation modifies innate immune cell migration and homing. Radiotherapy is known to induce immune-mediated anti-cancer responses through engagement in the cancer-immunity cycle, which includes a cascade of events: antigen presentation by DCs, T-cells activation, trafficking and infiltration of CTLs to cancers, recognition of cancer cells and killing cancer cells (Wu et al., 2023). One study showed that cancer cells could promote DCs to capture more tumor antigens and present processed antigens to T cells (Teitz-Tennenbaum et al., 2008). Liu et al. found that radiation could suppress LPS-triggered upregulation of CCR7 expression and PGE2 (prostaglandin E_2_) production by DCs, resulting in impaired DC migration towards CCL19 (Liu et al., 2011). A study by Morganti et al. showed that (Morganti et al., 2014) a single dose of 10 Gy irradiation decreased the percentage of resident microglia and increased peripherally CCR2+ macrophages’ infiltration as well as changed hippocampi’s level of pro-inflammatory cytokines. In addition to DCs, radiotherapy also affects neutrophils and NK cells. A study found radiotherapy increased neutrophils and myeloid-derived suppressor cells (MDSCs) in the TME of human pancreatic ductal adenocarcinoma, and STAT3 phosphorylation induced by radiotherapy was associated with the increased MDSCs infiltration and proliferation (Oweida et al., 2021). Radiotherapy could trigger NK cell anti-cancer immunity for fighting hepatocellular cancer relapse through activating cGAS–STING pathway and immunogenic cell death (Gong et al., 2025). The cGAS signaling pathway plays key roles in linking the DNA damage response with the immune responses (Lian et al., 2025). We specifically describe the role of cGAS signaling pathway in the relationship of radiation and immune response in Section 3 below. In addition, invariant natural killer T cells (iNKT), a subset of innate lymphocytes, could recognize lipid antigens to mediate immune regulation through secretion of IFN-γ and IL-4 (Paget et al., 2007). Also, iNKT cells can activate DCs, and the two cell types together bridge innate and adaptive immune (Stirnemann et al., 2008). Radiotherapy increased the infiltration and activation of DCs but failed to recruit NK cells and CD8^+^ T cells in PDAC patient tissue samples (Piper et al., 2022). However other study showed reprogrammed cancer TME through increasing NK cells (Godfroid et al., 2025). The tumor vasculature can be restored following radiotherapy from an influx of circulating cells consisting primarily of bone marrow derived monocytes and macrophages. The radiation-induced influx of bone marrow derived cells (BMDCs) into tumors can be prevented through the blockade of various cytokine pathways and such strategies can inhibit tumor recurrence (Russell & Brown, 2013).
(2) Radiation alters innate immune cell activity, either activating or eliminating these cells. Radiation generates ROS and DNA damage that can induce several forms of cell death and stress responses. In some contexts, micronuclei or mitochondrial/nuclear DNA in the cytosol activate cGAS–STING and related pathways, leading to type I interferon and inflammatory cytokine production (McMahon et al., 2023). In cancer cells, radiotherapy reprogrammes the TME, activating DNA and RNA sensing cascades, which in turn promote adaptive immunity (Wang, Kang, et al., 2024). Radiation-induced DNA damage can generate micronuclei or cytosolic DNA fragments. Cytosolic DNA sensors such as cGAS and AIM2 can then detect aberrant DNA and activate STING-dependent interferon signaling or inflammasome pathways, respectively (Cai et al., 2014; Hu et al., 2016). These signals culminate in type I interferon production and in the recruitment and activation of antigen-presenting cells (Hayman & Glazer, 2021). Liu et al. identified a novel mechanism by which innate immune response supports DNA damage repair via guiding synthesis of deoxyribonucleotide, the building blocks for DNA repair. The cGAS–STING–TBK1 axis is required for radiation-induced phosphorylation and activation of phosphoribosyl pyrophosphate synthetase 1/2 (PRPS1/2). The activated PRPS1/2 activation catalyses deoxyribonucleotide synthesis and promotes cells viability. Inhibition of the cGAS–STING axis disrupts radiation-induced PRPS1/2 activation and impairs cancer cell DNA repair and viability. This study highlighted an innate immune response-mediated DNA repair mechanism in cancer cells post-radiation (Liu et al., 2021). Radiotherapy, particularly when delivered to bone-marrow-containing regions and combined with chemotherapy, can cause acute hematologic toxicity, including leukopenia and neutropenia (Hui et al., 2014). In patients with hepatocellular carcinoma receiving radiotherapy, circulating CD14+HLA-DR-/low myeloid-derived suppressor cells changed during treatment and carried prognostic significance (Wang et al., 2016).
(3) Radiation alters cytokine secretion profiles. Ionizing radiation can selectively alter cytokine production by DCs. Malecka et al. reported that 1–6 Gy irradiation markedly suppressed IL-23 secretion, modestly decreased IL-12p70, and did not affect TNF-α, IL-1β, IL-6, IL-10, or IL-27 secretion (Malecka et al., 2016). Indeed, SASP (senescence-associated secretory phenotype) is also associated with altered cytokine profiles. A study used senescence-related exosomes to treat marrow-derived mesenchymal stem cells and found TGF-β elevated (Lee et al., 2023). Radiation could induce cellular senescence of human mesenchymal stem cell with increased levels of cytokine factors including IL-8 (Wang & Jang, 2009).

(4) Radiation-induced tumor cell killing is primarily mediated by DNA damage and cellular stress responses, while innate immune cells can modulate subsequent antitumor immunity and treatment outcome. Several studies have shown that radiation modulates the cytotoxicity activity of innate immune cells. Kim et al. reported that effective anti-cancer activity is increased via the sensitized DCs (maturation and activation) with γ-irradiated apoptotic cancer cells, enhancing cytotoxicity both directly and through indirect activation of T cells (Kim et al., 2013).

These signaling alterations form the fundamental basis of how radiation influences the innate immune response, as numerous chemical and immunological reactions occur post-radiation. Indeed, radiation exerts both immune-activating and immunosuppressive effects, which may depend on different radiation doses and varying biological contexts (McMahon et al., 2023). A deeper understanding of how radiotherapy impacts the innate immune system is essential for optimizing radio-immunotherapy combinations.

Macrophages illustrate how sharply the direction of the innate response depends on dose. Doses in the range of 0.5 to 2 Gy program tumor-associated macrophages toward an iNOS-positive M1 phenotype, normalize the tumor vasculature and license the entry of effector T cells, an effect that has been reproduced in pancreatic and other transplantable models (Klug et al., 2013). Higher single doses tend instead to enrich M2-like macrophages and myeloid-derived suppressor cells that restrain the same response, and host STING signaling contributes to this suppressive arm by mobilizing myeloid-derived suppressor cells through CCR2 (Lin et al., 2021; Wang et al., 2022) The pathway that produces interferon after irradiation therefore also seeds extrinsic radioresistance when it is chronically engaged, which is one reason that simply increasing the strength of innate activation is not a reliable therapeutic strategy.

Extracellular vesicles provide a second and spatially extended channel for the same signals. Exosomes released by irradiated cancer cells carry TREX1-sensitive double-stranded DNA to DCs, where they trigger STING-dependent interferon production, upregulation of costimulatory molecules and protective CD8-positive T cell responses, so that the radiation signal is transferred to cells that were never irradiated (Almars et al., 2025; Diamond et al., 2018). Because vesicle cargo also includes tumor antigen, immunosuppressive cytokines and PD-L1 itself, the net effect of vesicle traffic after irradiation is context dependent, and the cargo composition rather than the vesicle count is the informative variable (Almars et al., 2025).

### 2.5 Adaptive immune response to ionizing radiation damage

The adaptive immune response, which may contribute to the development of tailored radiotherapy strategies, is considered a critical component of precision multi-modality cancer treatment (Coleman et al., 2020). Adaptive immunity comprises T-cell-mediated cellular immunity and B-cell/antibody-mediated humoral immunity (Yu et al., 2023). Cellular immunity plays a crucial role in regulating the anti-cancer response through antigen recognition, T-cell activation, and immune-cell infiltration (Wang, Qi, et al., 2023). Radiation-induced DNA damage can generate cytosolic nucleic acids and immunogenic tumor material. Nucleic-acid sensing and DAMP-driven maturation promote dendritic-cell activation, after which DCs can cross-present tumor antigens, including neoantigens where present, to prime adaptive T-cell responses (Frey et al., 2012). Radiation can induce interferon-dependent PD-L1 expression on tumor cells in some settings, creating an adaptive immune-resistance mechanism. Actually, PD-L1 upregulated by radiotherapy maybe a cancer immune evasion mechanism through suppressing T lymphocyte proliferation and cytokine secretion leading to radiotherapy resistance (Hou et al., 2024). Inhibition of PD-L1 could overcome radio-immunotherapy resistance in lung cancer (Yi et al., 2024). Combination of radiotherapy and anti-PD-L1 antibody could enhance anti-cancer effect and improve radiation-induced resistance in non-small cell lung cancer (Gong et al., 2017). These findings indicate that both innate and adaptive immune responses are influenced by radiotherapy. Following radiation exposure, T cells are activated, and tumor-associated antigens bind to major histocompatibility complex class I (MHC I) molecules, which are then presented to CD8^+^ T lymphocytes. CD8^+^ T cells are direct cytotoxic T cells against target cells. There are several subsets depending on their cytokine profile, known as the cytolytic cytokine-producing Tc1, Th2 cytokines-producing Tc2, IL-9-producing Tc9, IL-17-producing and STAT3-and RORγ-expressing Tc17, IL-22-producing Tc22, and CXCR5-expressing Tfc cells. CD8-positive T cells can also differentiate into effector and memory populations distinguished by markers such as CD62L, CCR7, CD45RA, CD45RO, CD27, and CD28 (Decrion et al., 2007). Notably, it has been reported that CTLA4i could enhance CD4^+^ T helper cells expansion whereas radiotherapy promotes the recruitment of CD8^+^ T cells (Rudqvist et al., 2023). In patients with esophageal adenocarcinoma, radiotherapy promoted T cell function via increasing IFN-γ levels by CD4^+^ helper cells and increasing CD8^+^ T cell cytotoxic potential (Davern et al., 2024).

The balance between effector and regulatory T cells is the second determinant of the adaptive response, and it shifts in an unfavorable direction after irradiation unless it is deliberately managed. Regulatory T cells are comparatively radioresistant, increase in relative frequency within the irradiated field, and are functionally more suppressive than regulatory T cells recovered from unirradiated tumors (Balogh et al., 2013; Persa et al., 2015). In head and neck models, resistance to combined radiotherapy and PD-L1 blockade tracked with upregulation of TIM-3 and with regulatory T cell infiltration, and was reversed either by adding TIM-3 blockade or by depleting regulatory T cells (Oweida et al., 2018). Effector cells are not uniformly vulnerable, however. Tumor-reprogrammed resident memory T cells resist doses that eliminate circulating lymphocytes and can sustain control of an irradiated lesion, which indicates that the relevant question is not how many lymphocytes survive but which subsets do (Arina et al., 2019). This asymmetry supplies a clear rationale for pairing radiotherapy with agents that deplete or destabilize regulatory T cells, and it is the reason that CTLA-4 blockade and radiotherapy act through non-redundant mechanisms (Wang, Lynch, et al., 2024; Frijlink et al., 2024).

### 2.6 The abscopal effect: local irradiation with systemic consequences

Local irradiation occasionally causes regression of tumors that were never inside the treatment field. The potential mechanism underlying this effect may be that low-dose radiation increases the secretion of chemokines such as CXCL9 and CXCL10, attracting T cells and allowing CD8^+^ T cells to migrate to distant tumor sites (Sahin et al., 2025). This implies that low-dose radiation can amplify distant immune effects. Additionally, multiple studies have found that low-dose radiation can reshape the TME by increasing T-cell and NK-cell populations, polarizing macrophages toward the M1 phenotype, and achieving cancer control via immunostimulatory factors such as NK group 2 member D (NKG2D) (Herrera et al., 2022). This phenomenon, in which a local treatment elicits regression of distant, non-irradiated metastases, is termed the abscopal effect (Dotsu et al., 2026; Goh et al., 2023). A leading proposed mechanism is that local radiotherapy can, in some settings, contribute to a systemic antitumor immune response. The key steps are as follows: radiation can directly kill tumor cells, leading to immunogenic cell death. This form of cell death can release or expose tumor-associated antigens, including neoantigens when present, together with danger-associated molecular patterns such as ATP and HMGB1 that promote antigen presentation. Antigen-presenting cells, mainly DCs, are then activated. DCs engulf these tumor antigens, process them, and then migrate to nearby lymph nodes. In lymph nodes, activated DCs present processed tumor antigens to cytotoxic T cells that can specifically recognize and attack all tumor cells carrying the same antigen. These activated and amplified specific T cells migrate to various parts of the body through the bloodstream. They not only identify and attack the original tumor lesions that have received radiotherapy, but also search for and attack metastatic tumor lesions that have not been irradiated in other parts of the body and express the same antigen, resulting in distant lesions shrinking (Frey et al., 2012; Chen, Levy, et al., 2025). Chen et al. reported that low-dose irradiation of the gut improved responses to PD-L1 blockade in metastatic cancer patients. They reported this abscopal effect of low-dose radiation depended on dosimetry and immune host-microbiota, which can activate CD8^+^ T cell without exhaustion. Furthermore, intestinal christensenella minuta can enhance anti-tumor efficacy of low-dose radiation and PD-L1 blockade, leading to emigration of intestinal PD-L1-expressing DCs to cancer-draining lymph nodes (Chen, Levy, et al., 2025).

Several treatment-design variables, including dose per fraction, nodal irradiation and myeloid or cytokine support, can influence the probability of an abscopal response, but tumor-intrinsic and host immune factors also strongly constrain whether such a response occurs. The first is fraction size. Fractionation can influence systemic immune responses. The second is the integrity of antigen transport to the draining lymph node. Irradiated cancer cells package interferon-stimulatory DNA into exosomes that reach DCs (Diamond et al., 2018), and elective irradiation of tumor-draining lymph nodes abolishes the benefit of combining stereotactic radiotherapy with checkpoint blockade, which means that nodal target volumes are an immunological as well as an oncological decision. The third is the availability of a myeloid partner. Granulocyte-macrophage colony-stimulating factor combined with local radiotherapy generated abscopal responses in a proof-of-principle trial in patients with metastatic solid tumors (Golden et al., 2015), and radiotherapy has likewise been reported to enhance responses of lung cancer to CTLA-4 blockade (Wilkins et al., 2019).

## 3 Molecular interaction networks as the interface between radiation and immunity

Immunotherapy has emerged as a transformative treatment paradigm for numerous malignancies, and in many ways, this era is defined by the advancements in cancer immunotherapy (Chuprin et al., 2023). However, the molecular mechanisms underlying cancer immunotherapy are highly complex, involving numerous factors and molecules that regulate therapeutic responses. Interactions among immune-cell subsets, cytokine signaling and microbiome-associated factors may influence treatment outcome. For instance, the lung TME is a highly dynamic environment composed of various cells, molecules, and microbiota, all of which contribute to cancer development, progression, and resistance to radiotherapy and immunotherapy. Saeed Issa et al. developed a complex interaction model that illustrates the interplay between cancer cells and various factors within the lung TME (Saeed Issa et al., 2025). In this section, we examine how radiation reshapes these interactions and how the resulting network state constrains the response to immunotherapy. Understanding these processes and recent findings could facilitate the rational design of improved immunotherapies targeting cancer-associated antigens (Ghosh et al., 2025). We define molecular interactions as the interactions between genes, proteins, and drugs, along with gene and protein expression and functional annotation results, such as genetic interactions, PPIs, protein–DNA interactions, and gene– or protein–drug interactions with immunotherapy-targeted drugs.

### 3.1 Radiation as a dynamic regulator of molecular interaction networks

Radiotherapy is still frequently described as a cytotoxic agent that happens to have immunological side effects. That description is no longer adequate. A more useful formulation is that ionizing radiation both kills tumor cells and dynamically regulates molecular interaction networks connecting DNA damage responses to antitumor immunity; the behavior of combined regimens depends on both cytotoxic and immune-network effects. We therefore organize the mechanistic argument of this review around a three-tier axis. In the first tier, radiation physically disrupts protein–DNA contacts and forces the assembly of new protein complexes at damaged chromatin. In the second tier, the products of that disruption are detected by sensing complexes whose output is transcriptional. In the third tier, that transcriptional output determines the composition and functional state of the tumor immune microenvironment, and therefore the response to immunotherapy. Each tier corresponds to a distinct class of molecular interaction, and each supplies a different kind of therapeutic target.

The first tier begins within minutes of exposure. Double-strand breaks recruit the MRE11-RAD50-NBS1 complex, which activates ATM, while resected single-stranded ends engage ATR through RPA and ATRIP. Both kinases phosphorylate histone H2AX across megabase domains, and γ-H2AX foci and chromosomal aberrations are commonly used readouts of radiation-induced DNA damage and repair capacity (Nishibuchi & Tashiro, 2024). This is a protein–DNA and protein–protein interaction problem in the strict sense, because the outcome is decided not by the abundance of any single factor but by which complex occupies the break first. Its relevance to immunotherapy is direct, since repair choice determines how much unrepaired DNA eventually reaches the cytoplasm. Fan et al. showed that a radiation-induced interaction between ZNF131 and BACH1 protects both proteins from CUL3-mediated degradation and accelerates RAD51-dependent homologous recombination, and that disrupting this single interaction restores radiosensitivity in non-small-cell lung cancer (Fan et al., 2024). Chromosome segregation errors after irradiation also generate micronuclei whose fragile envelopes rupture during the following interphase, exposing genomic DNA to the cytosol and providing the substrate for the second tier.

In the second tier, three signaling modules dominate, and all three are amenable to pharmacological control. The cGAS–STING module is the best characterized. Cytosolic double-stranded DNA is bound by cGAS, which synthesizes 2′3′-cGAMP, activates STING at the endoplasmic reticulum and drives TBK1-dependent phosphorylation of IRF3 and the production of type I interferon. Deng et al. showed that STING, and not MyD88, is required for the type I interferon-dependent antitumor effect of radiation, and that DCs are the critical STING-expressing compartment (Deng et al., 2014). The module carries its own brake, since fraction sizes above roughly 12 Gy induce TREX1 and remove the ligand before cGAS can engage it. Cheng et al. reported that radiation induced IFN-α and CXCL9 expression in A549 cells, leading to CD8^+^ T cell migration (Cheng et al., 2021). The JAK–STAT module operates largely downstream of the interferons produced by the first two. In melanoma-bearing mice, carbon-ion irradiation was reported to reduce MDSC populations through a JAK2/STAT3-dependent mechanism and to increase antitumor immune infiltrates (Zhou et al., 2021). TGF-β, activated within the irradiated field, acts across all three modules by suppressing cross-presentation and restraining the priming of new T cell clones (Farhood et al., 2020).

The third tier is where these molecular events acquire clinical meaning. Because PD-L1 induction after irradiation is an interferon-driven adaptive resistance mechanism rather than a random consequence of damage, it is predictable and therefore exploitable. Continuous PD-L1 blockade during a fractionated course prevented the acquired resistance that otherwise developed, whereas blockade started after the course did not (Dovedi et al., 2014). In breast-cancer models, irradiation increased extracellular-vesicle-associated TGF-β1 and Treg infiltration, and targeting the radiation-activated PKC-ζ–TGF-β1EV pathway improved radiotherapy efficacy (Zhang et al., 2023). Radiation combined with CTLA-4 blockade expands T cell receptor clonotypes that neither treatment expands alone, which indicates that the combination changes the quality and not only the quantity of the antitumor repertoire (Twyman-Saint Victor et al., 2015). The practical inference is that dose, fractionation, target volume and timing should be selected for their effect on this network, and not only for their effect on the irradiated cells.

Framed in this way, the taxonomy of molecular interactions used in the remainder of the section is not a catalog but a set of intervention points. Protein–protein interactions govern complex assembly at damaged chromatin, nucleic-acid sensing and the formation of the immune synapse. Protein–DNA interactions govern repair choice and the transcriptional programs that follow. Fig. 1 summarizes the taxonomy together with the three-tier axis, Fig. 2 presents the corresponding mechanistic flowchart, and Fig. 3 summarizes the dose- and time-dependent immune-inflammatory dynamics that follow irradiation.

**Fig. 1:**
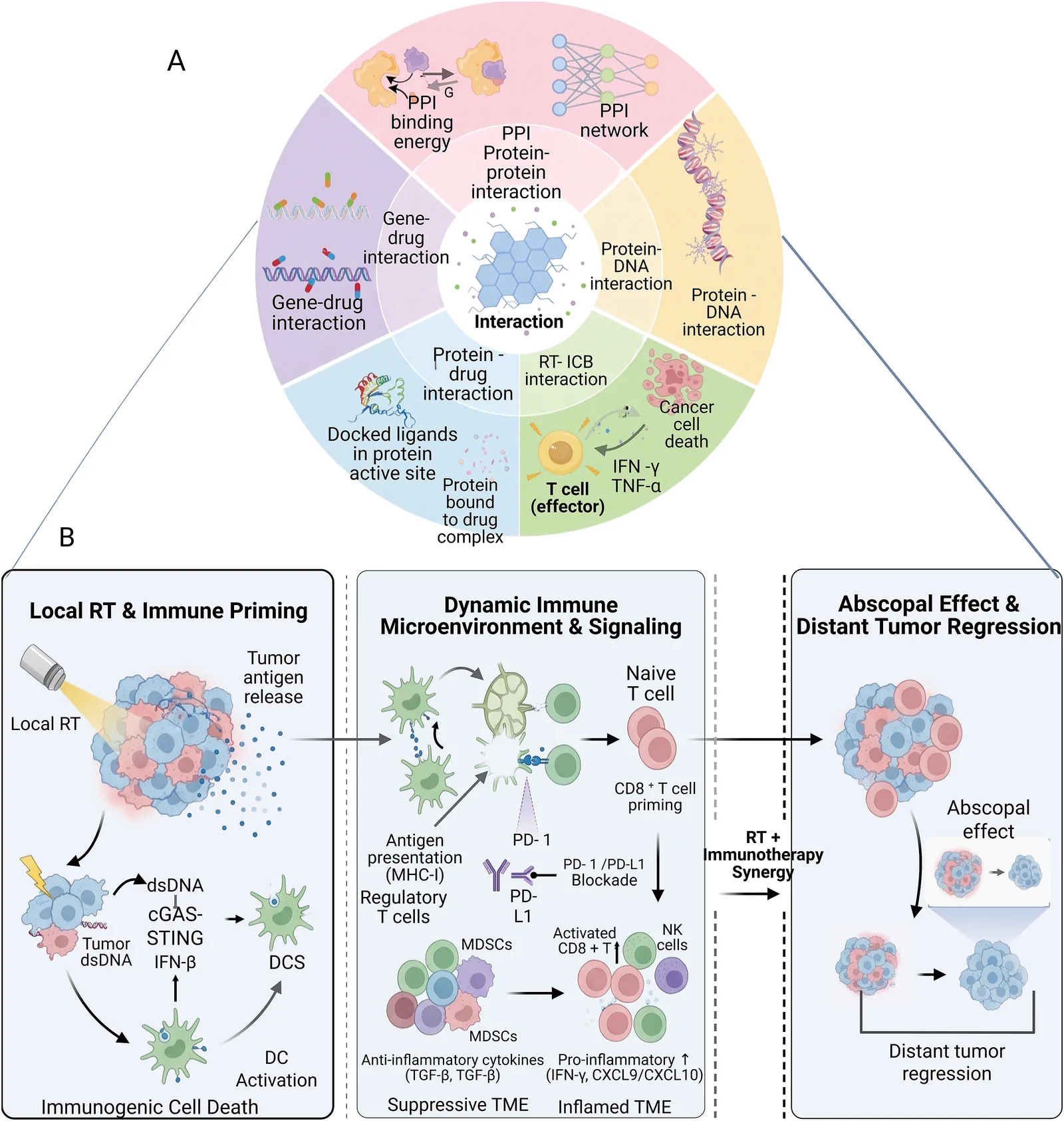
**Classification of molecular interactions and an integrative mechanism of radiotherapy-immunotherapy synergy.** A detailed and holistic conceptual framework for understanding radiotherapy-immunotherapy synergy. Panel (A) establishes a structured taxonomy of the primary molecular interaction types, including protein–protein, protein–DNA, and gene–drug interactions, to define the relevant molecular dictionary. Panel (B) presents an evidence-based, integrative mechanistic flowchart detailing the dynamic interplay from local radiotherapy to systemic, distant tumor response. It illustrates the critical transition from a suppressive to an inflamed microenvironment, specifically highlighting how PD-1/PD-L1 blockade occludes checkpoint signaling. This may enhance antigen presentation and T-cell priming, thereby increasing the likelihood of systemic antitumor immune responses, including occasional abscopal responses and distant tumor regression in selected contexts. RT, radiotherapy.

**Fig. 2:**
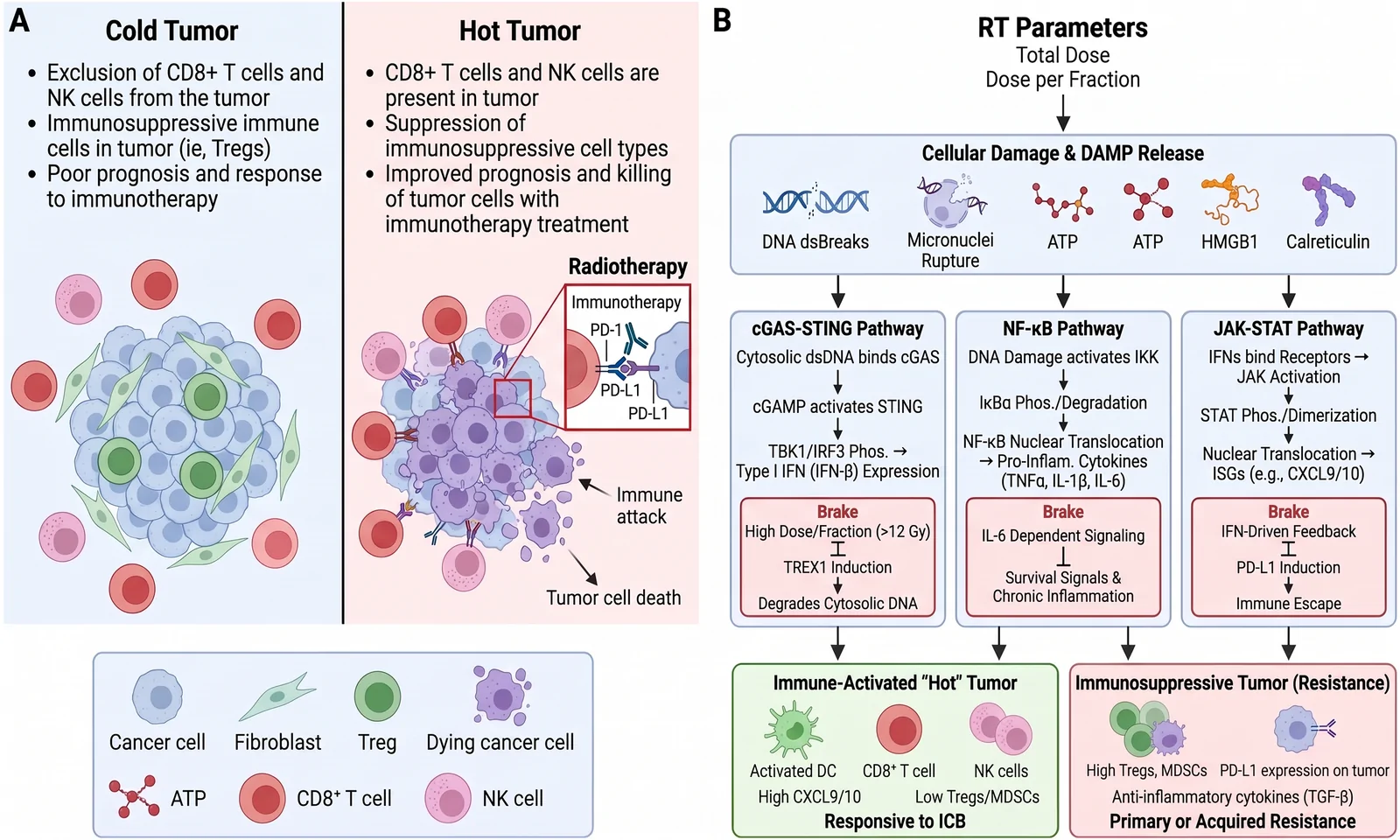
**Radiotherapy-mediated reprogramming of the tumor immune microenvironment.** (A) Comparative overview of immunologically cold vs hot tumor phenotypes, illustrating the role of radiotherapy in converting cold tumors to hot states and potentially improving immunotherapy outcomes. (B) A detailed molecular mechanistic flowchart showing how radiotherapy-induced cellular damage activates the cGAS–STING, NF-κB, and JAK–STAT pathways. Each pathway’s cascade and associated molecular brakes are depicted. The convergence of these signals determines the transition to either an immune-activated “hot” tumor responsive to ICB or an immunosuppressive tumor with primary or acquired resistance. RT, radiotherapy.

**Fig. 3:**
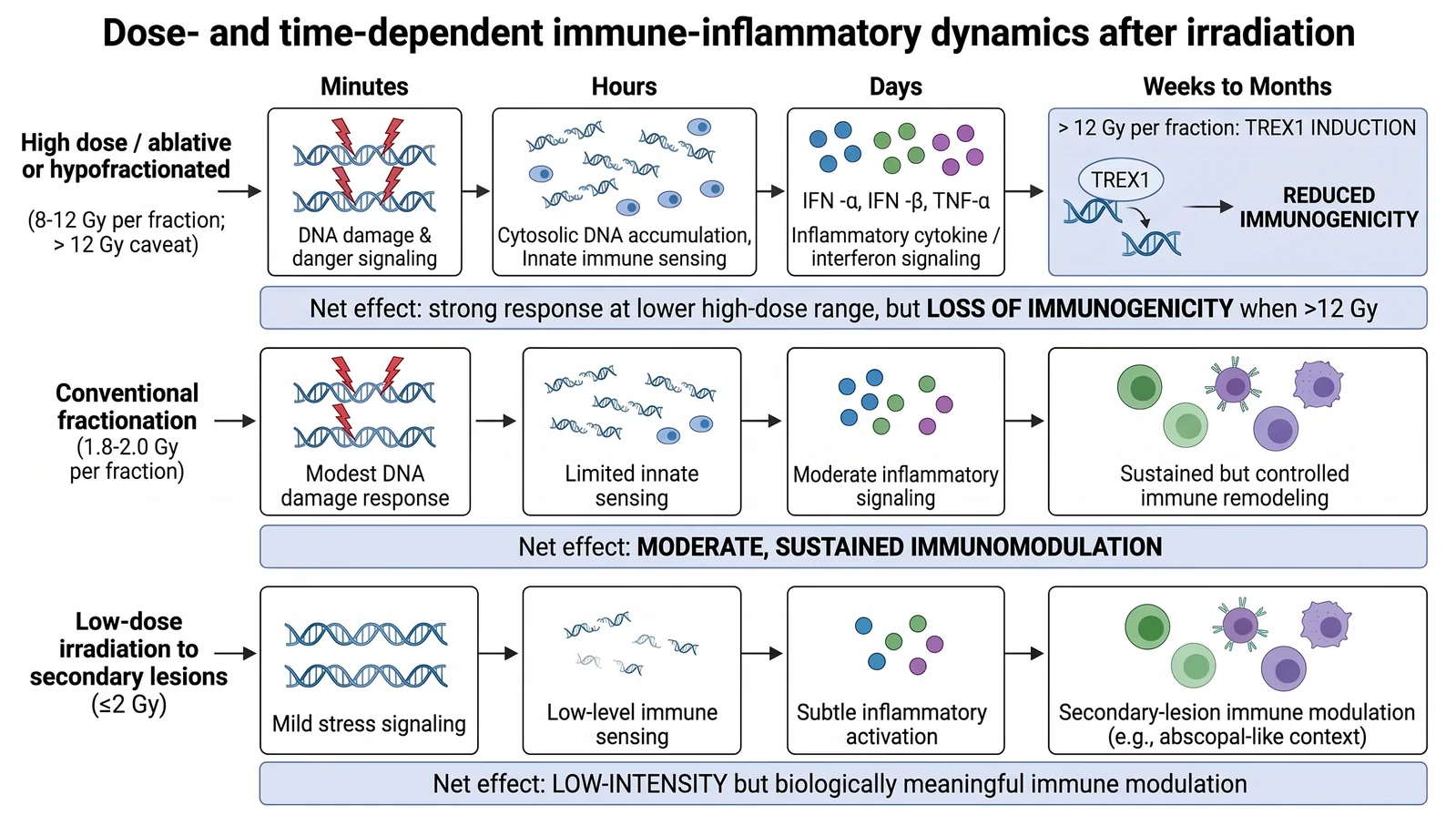
**Dose- and time-dependent immune-inflammatory dynamics after irradiation.** This matrix details the spatiotemporal evolution of immune responses across three radiation regimens: high-dose/hypofractionated, conventional, and low-dose. It traces the biological cascade from initial DNA damage (minutes) to long-term immune remodeling (weeks/months). Preclinical studies suggest that sufficiently high single-fraction doses can induce TREX1 in a dose- and context-dependent manner, promoting cytosolic DNA degradation and potentially attenuating cGAS–STING-dependent immunogenicity; the clinical threshold and consequences remain context dependent.

### 3.2 Protein**–**protein interaction (PPI)

Since the discovery of the first protein structure in 1958, the rapid advancement of protein identification techniques, including high-throughput screening and cryo-electron microscopy, has significantly accelerated the study of PPIs. PPI forms a large and complex intricate cellular network to orchestrate cell signal transduction, gene expression, homeostasis, and response to external stress (Afonso et al., 2025). In cancer cells, PPI is aberrant and associated with cancer formation and proliferation (Afonso et al., 2025). For much of the 20th century and the early molecular-targeted era, drug discovery often emphasized individual targets, whereas systems biology and interactome approaches later highlighted network-level mechanisms. It has been difficult to capture the complexity of biological systems (Chen, Yang & Hua, 2025) since “No organ system is an island” (Wen et al., 2024) and even multiple proteins conduct their functions in a form of complexes through PPI instead of working alone. Therefore, the emergence of PPI research marked a turning point, in that it considers the cell response activities as an entire and complex system (Chen, Yang, et al., 2025). PPI study also promotes the development of omics technical and the combination of radiotherapy and immunotherapy. Moreover, the difference of PPI from previous targeted therapy is that peptide-based inhibitors showed more promising outcomes with improved specificity and enhanced targeted binding ability, highlighting peptide-based inhibitors based on PPI study have potential serving as therapeutic agents (Afonso et al., 2025). These developments have facilitated the identification of PPI modulators, biomolecular structures, and their potential functions in radiotherapy and cancer immunotherapy (Nada et al., 2024). Further research into PPI functions in structural support, catalysis, signaling pathway transduction, and molecular cell membrane transport is expected to enhance targeted therapy achievements in cancer treatment (Banani et al., 2017). Indeed, the next decade may be regarded as the era of PPIs, with the transition from PPI research to clinically validated targets becoming an exciting reality.

Two features of protein–protein interactions matter specifically in the irradiated tumor. The first is that irradiation changes the interactome rather than simply the proteome. Complexes that are absent from resting cells, such as the 53BP1-RIF1-shieldin module at a resected break or cGAS bound to chromatin on a ruptured micronucleus, assemble only after damage, and they are therefore accessible to inhibitors within a defined therapeutic window rather than continuously. The second is that the central axis of checkpoint blockade is itself a protein–protein interaction. PD-1 engagement by PD-L1 is a low-affinity and rapidly reversible contact at the immune synapse, and approved PD-1 and PD-L1 antibodies block the PD-1–PD-L1 interaction, whereas other checkpoint antibodies, such as CTLA-4- or LAG-3-targeting agents, interrupt distinct receptor–ligand interactions. Because irradiation raises PD-L1 density through the STAT1 and IRF1 axis described above, radiation and checkpoint antibodies act on opposite sides of the same interaction, which is the most concrete molecular statement of their synergy that the current evidence supports.

### 3.3 Protein–DNA interaction

Regarding protein–DNA interactions, they could occur during the assembly of advanced structures and physiological functions within cells (Dickey et al., 2013). Protein–DNA interaction plays a crucial role in many important life processes, including DNA replication, transcription, damage repair, and gene expression regulation (Anashkina, 2023; Szymczyk et al., 2025). Rather than reviewing these binding chemistries in general terms, we focus on the three protein–DNA interfaces that are actually remodeled by radiotherapy and that carry consequences for immunotherapy. The first is the damage-sensing interface. Within seconds of irradiation, the MRN complex, the Ku70/Ku80 heterodimer and PARP1 bind broken DNA ends and help bias repair pathway choice; together with cell-cycle state, chromatin context, end processing and nuclease activity, these events can influence how much damaged or missegregated DNA later becomes available for cytosolic sensing. The second is the cytosolic sensing interface. cGAS activation is influenced by double-stranded-DNA binding, DNA length and oligomerization, while micronuclear DNA and TREX1-mediated DNA degradation can regulate radiation-induced cGAS–STING signaling. The third is the transcriptional interface. Radiation-induced DNA-damage and innate immune signaling can alter the activity and chromatin occupancy of NF-κB, STAT1-associated interferon-responsive factors and IRF3, thereby contributing to chemokine and cytokine programs such as CXCL9/CXCL10, IL-6 and TGF-β; the final secretory phenotype depends on multiple cell-intrinsic and microenvironmental variables. In each case the therapeutic question is not how tightly a protein binds DNA but which protein occupies the site first, and for how long, so that agents which shift this competition, including ATM and ATR inhibitors, PARP inhibitors and TREX1 inhibitors, act as immunological modifiers as much as radiosensitizers.

## 4 From molecular interactions to therapy: targeting strategies and the radiotherapy-immunotherapy interface

Cancer immunotherapy includes several therapeutic classes, such as immune checkpoint inhibitors, adoptive cell therapies, cancer vaccines, cytokine-based therapies, oncolytic viruses and T-cell-engaging antibodies. Since the introduction of the first tumor immunotherapy drug, IFN-α, the field has rapidly evolved. Today, several immune checkpoint inhibitors—including PD-1/PD-L1 and CTLA-4 inhibitors—along with CAR-T therapy, cancer vaccines, and oncolytic viruses, have received regulatory approval and are continually expanding treatment options. In December 2013, *Science* designated tumor immunotherapy as “Breakthrough of the Year” (Couzin-Frankel, 2013). Further solidifying its significance, the 2018 Nobel Prize in Physiology or Medicine was awarded for groundbreaking research in immune checkpoint blockade, highlighting the transformative impact of immunotherapy in cancer treatment. These achievements have significantly contributed to the expansion of the tumor immunotherapy drug market. With the continued development and integration of targeted therapies, molecular interaction studies, and advanced immunotherapeutic approaches, this market has continued to expand.

### 4.1 Converting interaction maps into druggable nodes: gene**–**drug and protein**–**drug interactions

The interaction networks described in Section 3 are useful to the extent that they nominate targets, and three operations connect the two. Target nomination identifies nodes whose removal changes the behavior of the network rather than merely its composition; in irradiated tumors these are typically hubs at the junction of damage signaling and immune signaling, such as STING, TREX1, TGF-β and the PD-1 and PD-L1 pair. Druggability assessment then asks whether a nominated node presents a surface that a small molecule or a biologic can occupy, a question that structure prediction has made tractable at scale and that has moved protein–protein interfaces, once regarded as undruggable, into routine screening, as the examples below illustrate. Pharmacogenomic annotation, finally, asks whether the response to the resulting agent will be uniform across patients. The remainder of this subsection treats the last two operations, which correspond to protein–drug and gene–drug interactions.

Study on protein–drug interaction plays important role in understanding drug mechanism, exploring novel drug targets as well as facilitating cancer treatment (Zheng et al., 2024). Many drugs exert their effects by reversible noncovalent binding to protein targets, while a smaller subset forms covalent interactions; binding strength, residence time and functional consequence vary widely (Zheng et al., 2024). An interesting example was reported that antibody– or protein–drug conjugates typically consist of a tumor-recognition moiety, such as an antibody or ligand, linked to a cytotoxic payload through a chemical linker. They could interact with cancer markers in selective, strong and stable way for improving cancer therapy efficacy (Porębska et al., 2023).

Accordingly, several landmark studies have reported the clinical application of PPI modulators. The BCL-2 inhibitor venetoclax has been approved for the treatment of hematological malignancies, including acute myeloid leukemia (Sheth et al., 2024). By occupying the BH3-binding groove of BCL-2, venetoclax displaces pro-apoptotic partners and reactivates the intrinsic apoptotic programme in cells that depend on this interaction for survival. Sarilumab, targeting IL-6 receptor, is used for the treatment of rheumatoid arthritis (Onishi et al., 2025), and adagrasib (a covalent inhibitor of the KRAS G12C mutant) for therapy targeting *KRAS* (G12C)-mutated non–small cell lung cancer, including after sotorasib-related hepatotoxicity (Luo et al., 2024). These agents illustrate distinct modes of target engagement: venetoclax disrupts a BCL-2-family interaction, sarilumab blocks IL-6 receptor signaling, and adagrasib covalently inhibits mutant KRAS G12C. Their approvals do not by themselves establish efficacy in radio-immunotherapy combinations. Notably, with the advancements brought by AlphaFold, it is projected that the development of PPI-targeted radiotherapy and immunotherapy for cancer will rapidly accelerate (Zhu, Liu, Li, et al., 2023).

Regarding gene– or protein–drug interactions, it is commonly occurring in the drug discovery process (Pandey & Pandey, 2025). Through analysis of gene–drug interaction, it can provide researchers annotating known drug-gene interactions and potential genes associated with druggable accessibility (Wu, Chen, et al., 2024). Furthermore, the study of gene–drug interactions can guide dose adaptation and support more precise combinations of radiotherapy and immunotherapy (Türk et al., 2021). The occurrence of gene–drug is often to the drug-related pharmacology affected by genetic variation in drug-related metabolizing enzymes, transport genes (Türk et al., 2021). A well known example is that the CYP2C19, CYP2C9 and CYP2D6 polymorphisms link with many drugs’ metabolism (Ingelman-Sundberg, 2004). Candidate pharmacogenetic associations may be prognostic or treatment-associated, but predictive utility requires a validated treatment-by-biomarker interaction in independent cohorts. For example, patients with high-grade glioma with genotype of GSTP-1 polymorphism 313A>G had a worse prognosis when treated with radiotherapy plus temozolomide (Zhi et al., 2021). Therefore, identifying gene–drug interactions would help therapy adjustment to facilitate pharmacogenetic implementation in clinical therapy.

These examples share a design logic that transfers directly to radio-immunotherapy. Venetoclax was developed against a protein–protein interface rather than an enzymatic active site. Adagrasib exploits a covalent contact that exists only in a mutant conformation. The outcome of temozolomide combined with radiotherapy in high-grade glioma varies with a germline polymorphism in a detoxifying enzyme.

### 4.2 Cytokine immunotherapy

Cytokine-based immunotherapy was the earliest approach used in tumor immunotherapy, with IFN-α and IL-2 being the most widely applied in clinical practice. IFN-α enhances the activity of DCs and NK cells, strengthening the body’s anti-tumor immune response. In 1986, the FDA approved IFN-α as the first recombinant cytokine drug approved by the FDA for the treatment of hairy cell leukemia (Borden, 2019). Specific IFN-α products have held indications in selected malignancies, including hairy cell leukemia and AIDS-related Kaposi sarcoma; use in melanoma and other malignancies is product-, indication-, and jurisdiction-specific. Interleukins are produced by diverse immune and stromal cell types, including CD4^+^ helper T cells, macrophages, DCs, B cells, NK cells and tissue-resident cells, and they regulate CD8^+^ T cells, macrophages, NK cells and many other components of antitumor immunity (Briukhovetska et al., 2021). In 1992, recombinant human IL-2, produced using genetic engineering technology, was approved for the treatment of renal cell carcinoma and later for metastatic melanoma (Rosenberg, 2014). Contemporary use of IFN-α and high-dose IL-2 is limited to selected indications and patients (Borden, 2019; Rosenberg, 2014). Other cytokines are still in various stages of clinical research, with ongoing efforts to expand cytokine-based strategies for cancer treatment.

### 4.3 Antibody-based immunotherapy and immune checkpoint blockade

The FDA has approved several antibody-based immunotherapy drugs, including rituximab, ofatumumab, and alemtuzumab (Weiner et al., 2009). Daclizumab- or denileukin diftitox–based Treg-depletion strategies have been explored in early clinical studies, but antitumor efficacy and an acceptable benefit–risk profile have not been established for routine treatment of metastatic breast cancer or melanoma (Dannull et al., 2005). Ipilimumab, an anti-CTLA-4 antibody, and BRAF inhibitors such as vemurafenib modulate the melanoma immune microenvironment through distinct mechanisms (Chapman et al., 2011; Pardoll, 2012). Immune checkpoints are critical regulators of the body’s immune system, with stimulatory checkpoint molecules enhancing T-cell activation and immune response, while inhibitory checkpoint molecules act as natural “brakes” to prevent autoimmune reactions. Tumor cells exploit inhibitory immune checkpoints to suppress immune responses, enabling them to evade immune detection and destruction. To counteract this, antibody-based drugs have been developed to target common inhibitory immune checkpoints, blocking these interactions and enhancing immune system activity to eliminate tumors. To date, more than 10 immune checkpoints have been identified, with CTLA-4 and PD-1/PD-L1 being the most extensively studied (Pardoll, 2012). CTLA-4, a T-cell surface receptor, functions as an immunosuppressive molecule that transmits inhibitory immune signals. PD-1 inhibitors block the interaction between PD-1 on T cells and its ligands PD-L1/PD-L2, thereby relieving inhibitory signaling and restoring antitumor T-cell function. PD-1 inhibitors have been widely implemented in clinical practice and have demonstrated significant therapeutic success in various cancers.

### 4.4 Tumor vaccine immunotherapy

Tumor vaccines activate the body’s immune system to target and eliminate tumor cells. These vaccines mainly include DC vaccines, gene vaccines, and peptide vaccines (Saxena et al., 2021). DC vaccines, as exogenous antigen-presenting cells, capture tumor antigen information and present it to lymphocytes, thereby activating an immune response to kill tumor cells. Provenge is the first FDA-approved DC vaccine, primarily used for the treatment of metastatic castration-resistant prostate cancer (Saxena et al., 2021).

### 4.5 How radiotherapy potentiates immunotherapy

The strongest evidence concerns checkpoint blockade, and it is mechanistically specific rather than merely additive. Twyman-Saint Victor et al. showed that radiation, CTLA-4 blockade and PD-L1 blockade act through non-redundant mechanisms in melanoma: CTLA-4 blockade depletes regulatory T cells and raises the ratio of CD8-positive T cells to regulatory T cells, radiation diversifies the intratumoral T cell receptor repertoire, and PD-L1 blockade reverses the T cell exhaustion that limits both (Twyman-Saint Victor et al., 2015). Stereotactic irradiation increases cross-presentation of tumor antigen and expands antigen-specific T cells, an effect amplified by PD-1 blockade (Sharabi et al., 2015), and the clonotypes expanded by radiation combined with CTLA-4 blockade differ from those expanded by either treatment alone. In patients, adding radiotherapy to CTLA-4 blockade produced objective responses in chemorefractory metastatic lung cancer, with induction of IFN-β and expansion of T cell clones as immunological correlates (Formenti et al., 2018). Durvalumab consolidation after concurrent chemoradiotherapy is an established strategy for unresectable stage III NSCLC (Hui et al., 2019), and a pooled analysis of two trials suggested higher response rates and improved survival with radiotherapy plus pembrolizumab, including a signal in PD-L1-negative disease; this subgroup finding and its proposed antigen-release mechanism remain hypothesis-generating (Theelen et al., 2021).

Radiotherapy also changes what adoptive cell products can achieve. Low-dose radiation sensitized antigen-negative pancreatic tumor cells to TRAIL produced by locally activated CAR T cells, so that antigen-negative as well as antigen-positive cells were eliminated and antigen escape was reduced (DeSelm et al., 2018). In glioblastoma models, NKG2D-based CAR T cells and radiotherapy were synergistic because irradiation upregulates NKG2D ligands on tumor cells and thereby restores the target that the cell product requires (Weiss et al., 2018). In both settings radiation is not simply reducing tumor bulk. It is supplying the ligand or the death-receptor sensitivity on which the cell product depends, which is a mechanistic rather than a logistical form of synergy.

Innate agonists and delivery technologies act on the same axis from the opposite side. Radiation can generate cytosolic DNA that activates cGAS–STING in sensing-competent tumors; in colorectal cancer models, ATR inhibition with berzosertib has been reported to cooperate with irradiation by increasing cytosolic dsDNA, activating STING signaling and enhancing anti-PD-L1 therapy (Liu et al., 2023). Granulocyte-macrophage colony-stimulating factor added to local radiotherapy generated abscopal responses in a proof-of-principle trial (Golden et al., 2015). Antigen-capturing nanoparticles that collect proteins released from an irradiated tumor and carry them to draining lymph nodes improved responses to PD-1 blockade in melanoma models, and nanoscale metal-organic frameworks that enhance local dose deposition potentiated checkpoint blockade at both irradiated and distant sites. What these approaches share is that they treat radiotherapy as the antigen-release step of an in situ vaccine and then supply, separately, the adjuvant and the delivery vehicle that the vaccine requires.

### 4.6 How immunotherapy modifies tumor radiosensitivity

The reverse direction is discussed less often but is equally well documented. Lee et al. showed that the antitumor effect of ablative irradiation requires CD8-positive T cells and that depleting them converts a curative dose into a merely cytoreductive one (Lee et al., 2009). The corollary is important: measured radiosensitivity is in part an immunological property of the host rather than an intrinsic property of the tumor cell, so any intervention that enlarges or preserves the effector pool will appear, in the clinic, to have radiosensitized the tumor. The relative radioresistance of tumor-reprogrammed resident memory T cells provides a cellular basis for this effect and explains why some irradiated lesions remain controlled long after the irradiated tumor cells have been replaced.

Checkpoint blockade acts on the same pool. Continuous PD-L1 blockade during fractionated radiotherapy preserved the CD8-positive effector population, prevented the exhaustion phenotype that otherwise developed by the end of a fractionated course, and produced durable control that neither modality achieved alone (Dovedi et al., 2014). CTLA-4 blockade acts differently, by depleting regulatory T cells; because regulatory T cells are comparatively radioresistant and expand after irradiation (Balogh et al., 2013; Persa et al., 2015), removing them alters the effective dose-response relationship of the radiation itself. Neutralizing TGF-β during irradiation produces a comparable shift through a third route, by removing a brake on cross-priming rather than by changing the size of the effector pool (Farhood et al., 2020).

Two practical consequences follow. First, immunological radiosensitization is time dependent, so the interval between the two treatments is a design variable rather than a scheduling convenience; in murine models, anti-PD-1 given after stereotactic irradiation produced stronger abscopal responses than the same antibody given before it. Second, an immunological radiosensitizer fails if the radiation plan destroys the cells on which it depends. Elective irradiation of tumor-draining lymph nodes abolished the benefit of combining stereotactic radiotherapy with checkpoint blockade, and large-field or protracted regimens deplete circulating lymphocytes. Field design, and not dose alone, is therefore part of the immunotherapy.

### 4.7 A mechanism-based framework for combination design

The strategies described above can be organized by the mechanistic role that the partner agent plays relative to radiation, and four classes cover most of the current landscape. Innate immune agonists supply or amplify the sensing step, and they are appropriate when the tumor cannot generate an interferon response by itself. Adaptive immune enhancers act on the effector pool, and they are appropriate when priming occurs but is not sustained. Microenvironment remodeling agents remove suppressive constraints imposed by regulatory T cells, myeloid-derived suppressor cells or TGF-β, and they are appropriate when effector cells are present but non-functional. Delivery and targeting technologies change where the signal is generated rather than what the signal is.

## 5 Challenges and prospects

### 5.1 Clinical evidence and the badscopal effect

Clinical benefit from combining radiotherapy and immunotherapy is context-dependent: survival benefit is established in selected settings, whereas results in many other solid tumors remain mixed or investigational (Dagar et al., 2024). Immunotherapy and radiotherapy are closely interconnected—radiotherapy can increase tumor-antigen release and presentation, induce inflammatory danger signals, and in some contexts contribute to epitope spreading (Wu, Yi, et al., 2024). Additionally, radiotherapy reshapes the TME, potentially leading to a more durable and sensitive immunotherapy response. Thus, the integration of immunotherapy and radiotherapy represents a paradigm shift in cancer treatment. While some clinical trials have demonstrated promising results, such as in non-small-cell lung cancer, outcomes have been less encouraging in certain cancers like head and neck squamous cell carcinoma and colorectal cancer. Research indicates that intrinsic tumor heterogeneity may contribute to the differential responses observed with immunotherapy and radiotherapy. However, further studies on molecular interactions in combination therapies are essential for understanding treatment resistance mechanisms and improving therapeutic efficacy. Investigating molecular interactions can help identify new therapeutic targets to enhance both immunotherapy and radiotherapy. This could lead to the discovery of novel drugs or the development of strategies to overcome resistance to these treatments. Additionally, targeting key modulators responsible for regulating molecular interaction-related signaling pathways may provide deeper insights into the mechanisms underlying immunotherapy and radiotherapy resistance, ultimately paving the way for more effective cancer treatment strategies. A recent study of the early lung-metastatic microenvironment reported that radiotherapy-induced AREG promoted a cancer-tolerogenic phenotype in EGFR-expressing mononuclear phagocytes and induced STAT3-mediated CD47 upregulation in cancer cells. This revealed a previously underappreciated phenomenon, termed the “badscopal” effect, in which radiotherapy reshapes systemic immunity in a way that enhances metastatic outgrowth, indicating a need to develop therapeutic strategies targeting radiotherapy-induced immune modulation (Piffkó et al., 2025).

In some contexts, radiotherapy can increase antigen release, inflammatory signaling and immune infiltration in otherwise immune-“cold” tumors, potentially improving responsiveness to immunotherapy. However, combination therapy also presents challenges. Clinical studies have shown that combining PD-L1 (durvalumab) and CTLA-4 (tremelimumab) inhibition with radiotherapy did not significantly improve response rates in patients with non-small-cell lung cancer resistant to PD-L1 therapy (Schoenfeld et al., 2022). Additionally, radiotherapy may induce immunosuppressive effects, potentially limiting cancer control (McLaughlin et al., 2020). Lee et al. showed that the therapeutic effect of ablative irradiation depends on host CD8^+^ T cells; CD8^+^ T-cell depletion reduced tumor control after radiation, indicating an immune contribution to apparent tumor radiosensitivity (Lee et al., 2009). Phase III evidence supports post-chemoradiotherapy durvalumab consolidation in defined settings. Hui et al. reported patient-reported outcomes from the phase III PACIFIC trial in unresectable non-small-cell lung cancer. Their findings indicated durvalumab has a clinical benefit for patients after treating with chemoradiotherapy without compromising the patient-reported outcomes including cough, dyspnea, chest pain and so on (Hui et al., 2019). ADRIATIC showed that consolidation durvalumab after chemoradiotherapy improved survival outcomes in patients with limited-stage small-cell lung cancer (Addeo & Liu, 2024). Wei et al. conducted a series of mouse tumor experiments and found that compared to anti-PD-1 given prior to stereotactic body radiation therapy (SBRT), treating with anti-PD-1 after SBRT has more great benefits including antitumor immunity and protection results. This finding supposes that the timing of anti-PD-1 antibody administration in radiotherapy may influence the potency of relative abscopal response, which means the given order of immunotherapy is critical important (Wei et al., 2021). Table 1 summarizes clinical therapy outcomes from the combination of immunotherapy and radiotherapy. In Table 1, in lung cancer, using different radiation doses and various immunotherapy medicine, the OS and PFS results differed (Chang et al., 2023; Filippi et al., 2024; Li et al., 2023; Xu et al., 2025). However, there had study reported that in head and neck cancer, combination of 69.96 Gy in 33 fractions and Pembrolizumab (200 mg Q3W) did not improve the tumor control and survival but appeared less toxic (Tao et al., 2023). There was no significant difference in overall survival or progression-free survival in head and neck cancer patients treated with 9 Gy × 3 radiotherapy and nivolumab (McBride et al., 2021). Additionally, the results may be different within various cancer types. This may due to there exists different human genetic background or tumor heterogeneity, which need further investigation.

**Table 1:** Therapy outcomes from combination of immunotherapy and radiotherapy.

Cancer	NCT study ID	Study phase/design	No. of patients	Radiotherapy /radiopharmaceutical	Immunotherapy	OS/PFS	References
Lung cancer	NCT03798535	Observational (real-world)	1 154	60 Gy	Durvalumab (10 mg/kg every 2 weeks)	3-year OS rates were numerically higher among patients with PD-L1 expression on ≥1% vs <1% of tumor cells (TCs; 67.0% vs 54.4%).	Filippi et al. (2024)
Lung cancer	NCT03110978	II	156	Stereotactic ablative radiotherapy	Nivolumab (480 mg)	At a median 33 months’ follow-up, I-SABR significantly improved 4-year event-free survival from 53% (95% CI, 42%–67%) with SABR to 77% (66%–91%; per-protocol population, hazard ratio (HR) 0.38; 95% CI, 0.19–0.75; *P* = 0.005 6; ITT population, HR 0.42; 95% CI, 0.22–0.80; *P* = 0.008 0).	Chang et al. (2023)
Lung cancer	single-center retrospective study	-	36	52–113 Gy	Atezolizumab or durvalumab	The estimated 1-year OS was 80.2% and 1-year PFS was 53.4%.	Li, L. et al. (2023)
Lung cancer	NCT04291092	II	67	22–27 Gy	Camrelizumab (200 mg intravenously once every 3 weeks)	The 6-month progression-free survival rate was 71.7%.	Xu et al. (2025)
Head and neck cancer	NCT02707588	II	133	69.96 Gy in 33 fractions	Pembrolizumab (200 mg Q3W)	There was no significant difference between arms for progression-free survival (HR 0.85, 95% CI, 0.55–1.32; *P* =0 .47) and for overall survival (HR 0.83, 95% CI, 0.49–1.40; *P* = 0.49).	Tao et al. (2023)
Head and neck cancer	NCT02684253	II	62	9 Gy × 3	Nivolumab (3 mg/kg intravenously every 2 weeks)	No significant difference in overall survival (*P* = 0.75), progression-free survival (*P* = 0.79)	McBride et al. (2021)
Head and neck cancer	NCT03162731	I	24	70 Gy	Nivolumab (3 mg/kg every 2 weeks, 17 doses) plus ipilimumab (1 mg/kg every 6 weeks, 6 doses)	The 3-year PFS and OS rates were 74% (95% CI, 58%–94%) and 96% (95% CI, 88%–100%), respectively.	Johnson et al. (2023)
Pancreatic cancer	NCT02704156	II	147	35–40 Gy in five fractions	Pembrolizumab (200 mg)	For BED_10_ of 60–65 Gy, OS was similar between patients in the SBRT + K + M and SBRT + G groups (median, 13.6 vs 12.4 months; HR 0.69 (95% CI, 0.41–1.16); *P* = 0.16). For BED_10_ of ≥65 Gy, PFS was prolonged with SBRT + K + M vs SBRT + G (median: 8.6 vs 5.0 months, HR 0.48 (95% CI, 0.31–0.77); *P* = 0.002 1).	Zhu, Liu, Cao, et al. (2023)
Pancreatic cancer	NCT04258150	II	26	15 Gy	Nivolumab (6 mg/kg)	Median PFS and OS were 1.6 and 5.3 months.	Chen, Donia, et al. (2023)
Pancreatic adenocarcinoma	NCT04098432	I/II	15	4 × 8 Gy	Nivolumab (3 mg/kg intravenously every 2 weeks)	Median PFS and OS were 8.1 and 13.0 months.	Vošmik et al. (2024)
Pancreatic adenocarcinoma	Cohort study	-	96	54–63 Gy	Camrelizumab 200 mg once every 3 weeks	Median OS was 22.3 months; PFS was 12.0 months.	Chen, Li, et al. (2023)
Prostate cancer	NCT03093428	II	42	Radium-223 (R223)	Pembrolizumab (200 mg)	Median rPFS and OS was 6.1 (95% CI, 2.7-11.0) and 16.9 months (12.7–not reached (NR)), respectively, with R223+P and 5.7 (2.6–NR) and 16.0 (9.0–NR), respectively, with R223.	Choudhury et al. (2024)
Glioblastoma	NCT02667587	III	716	60 Gy over 6 weeks	Nivolumab 240 mg	median (m)PFS (blinded independent central review) was 10.6 months (95% CI, 8.9–11.8) with NIVO + RT + TMZ vs 10.3 months (95% CI, 9.7–12.5) with PBO + RT + TMZ (HR 1.1; 95% CI, 0.9–1.3) and mOS was 28.9 months (95% CI, 24.4–31.6) vs 32.1 months (95% CI, 29.4–33.8), respectively (HR 1.1; 95% CI, 0.9–1.3).	Lim et al. (2022)
Melanoma brain metastases	-	II	92	30–40 Gy	Nivolumab	patients starting with RT (Rad-ICI) for overall response rate (RR: *P* = 0.007; HR 7.88 (95% CI, 1.76–35.27)) and disease control rate (DCR: *P* = 0.036; HR 6.26 (95% CI, 1.13–34.71)) with a trend for a better PFS (*P* = 0.162; HR 1.64 (95% CI, 0.8–3.3)).	Hassel et al. (2022)
Advanced squamous cell cancer	NCT03686332	II	32	33 × 1.8 Gy	Atezolizumab (1 200 mg) once every 3 weeks.	Median OS was 11.3 months; 1-year PFS was 12.5%.	de Vries et al. (2023)
Colorectal cancer	NCT02992912	II	60	45 Gy	Atezolizumab (1 200 mg) once every 3 weeks	Median OS and PFS were respectively 8.4 and 1.4 months.	Levy et al. (2024)
Hepatocellular carcinoma	ChiCTR2200061793	multicenter single-arm prospective	30	52–56 Gy	Atezolizumab (1 200 mg) once every 3 weeks	Median OS was 9.8 months; the median PFS was 8.0 months.	Wang, Zhang, et al. (2023)
Hepatocellular carcinoma	a multicenter, open-label, noncomparative, randomized trial		60	36–40 Gy	Camrelizumab 200 mg	Median OS were 12.7 months (95% CI, 10.2–not available (NA)) and 8.6 months (95% CI, 5.6–NA), and median PFS were 4.6 months (95% CI, 3.3–7.0) and 2.5 months (95% CI, 2.0–7.6) for the SBRT and non-SBRT cohorts, respectively.	Hu et al. (2023)
Hepatocellular carcinoma	NCT04193696	A single-arm trial	21	30–50 Gy	Camrelizumab 200 mg	The overall survival probability was 85.7% at 6 months, 76.2% at 9 months, and 59.9% at 12 months.	Li, J. X. et al. (2022)
Hepatocellular carcinoma	-	II	30	45 Gy	Tislelizumab 200 mg	The ORR was 30.0% (90% CI, 16.6%–46.5%) and the median OS was 18.7 months. The median RFS was not reached with a median follow-up of 21.77 months (95% CI, 12.50–31.03) post-surgery.	Pan et al. (2024)
Hypopharyngeal carcinoma	NCT04156698	II	53	50.6 Gy	Camrelizumab 200 mg	The 2-year OS, PFS, and LPR rates were 83.0%, 77.1%, and 70.0%, respectively.	Gong et al. (2024)
Stomach cancer	NCT03631615	II	36	45 Gy	Camrelizumab 200 mg	2-year PFS and OS rates were 66.9% and 76.1%, respectively.	Tang et al. (2022)
Esophageal cancer	NCT04005170	II	42	50.4 Gy	Toripalimab 240 mg	1-year overall survival was 78.4% and 1-year progression-free survival was 54.5%.	Zhu, Wen, et al. (2023)
Nasopharyngeal carcinoma	NCT04398056	II	22	69.9–70.1 Gy	Toripalimab 240 mg	The ORR at 3 months after radiotherapy is 81.8% (22.7% complete response, *n* = 5; 59.1% partial response, *n* = 13), and the disease control rate is 81.8%. The 3-year progression-free survival (PFS) rate is 44.9% (95% CI, 26.4%–76.3%).	Chen, Duan, et al. (2023)
Nasopharyngeal carcinoma	NCT03854838	II	25	60–60.21 Gy	Toripalimab 240 mg	The 12-month PFS was 91.8% (95% CI, 91.7%–91.9%).	Hua et al. (2021)
Esophageal squamous cell cancer	ChiCTR2100045104	Ib	23	30 Gy	Toripalimab 240 mg	A 2-year PFS rate of 63.8% and 2-year OS rate was 78%.	Jiang et al. (2024)
Esophageal cancer	-	II	30	50 Gy	Tislelizumab 200 mg	1-year PFS and overall survival (OS) rates were 79.4% and 89.6%, respectively.	Wang, Lynch, et al. (2024)
Biliary tract carcinoma	NCT03892577	-	40	24–60 Gy	Toripalimab 240 mg	Median OS were 13.7 months; median PFS were 10.8 months.	Wang, Xiang, et al. (2023)
Melanoma	NCT01557114		19	9–24 Gy	10 mg/kg ipilimumab every 3 weeks for four doses	The median overall survival (95% CI) was estimated at 0.9 years (0.5–2). The median progression-free survival (PFS) (95% CI) was 0.4.	Boutros et al. (2020)

OS, overall survival; PFS, progression-free survival; Gy, gray; SBRT, stereotactic body radiation therapy; SABR, stereotactic ablative radiotherapy; I-SABR, immunotherapy plus SABR; ORR, objective response rate; DCR, disease control rate; RFS, recurrence-free survival; rPFS, radiographic progression-free survival; LPR, local progression rate; BED_10_, biologically effective dose (α/β = 10 Gy); HR, hazard ratio; RR, risk ratio; CI, confidence interval; ITT, intention-to-treat; NR, not reached; NA, not available; RT, radiotherapy; TCs, tumor cells; TMZ, temozolomide; NIVO, nivolumab; PBO, placebo; PD-L1, programmed death-ligand 1.

### 5.2 Mechanisms of resistance: evidence from classical preclinical models

Because the clinical picture is heterogeneous, the mechanistic account of resistance rests largely on a small number of transplantable murine models that have been used consistently across laboratories, and it is worth stating explicitly which conclusions come from which system. TSA and 4T1 are transplantable mammary carcinoma models that have been used repeatedly to study radiation-induced antitumor immunity. The MC38 and CT26 colorectal models are interferon-competent and were used to establish that STING is required for radiation-induced antitumor immunity and that host STING also drives myeloid-derived suppressor cell mobilization (Deng et al., 2014). The value of naming the models is that it makes the limits of extrapolation visible: transplantable tumors have high mutational load, short latency and intact lymphatic drainage, and none of these conditions reliably holds in treated patients.

Across these systems, resistance arises at three identifiable points, and each maps to a distinct intervention. It can arise at the sensing step when cGAS–STING signaling is epigenetically suppressed or when high single-fraction doses induce TREX1; potential strategies include altering fractionation to preserve cytosolic DNA signaling, using innate immune agonists when the relevant pathway remains functional, or restoring pathway competence where feasible. It arises at the priming step when tumor-draining nodes are irradiated or resected, when BATF3-dependent DCs are absent, or when TGF-β is active within the field, in which case the remedy lies in target volume design and in cytokine neutralization. It arises at the effector step when regulatory T cells and myeloid-derived suppressor cells expand, when inhibitory receptors such as TIM-3 are upregulated during PD-L1 blockade, or when circulating lymphocytes are depleted by large-volume irradiation (Oweida et al., 2018). Grouping resistance mechanisms by step rather than by tumor type is more informative for trial design, because it identifies which component of a failing regimen should be replaced.

The effectiveness of combining immunotherapy and radiotherapy in triggering remote immune effects is influenced by multiple factors. While numerous studies imply that this combination is promising and effective, the findings should be interpreted with caution, because significant differences exist between human and animal models. Additionally, there is a lack of reliable biomarkers for predicting immune responses to radiotherapy in clinical trials. Future human clinical studies should focus on tracking key immune cell populations throughout the radiotherapy process to understand treatment dynamics better. In this regard, the detection of circulating biomarkers presents a cost-effective and practical approach for predicting immune responses. These biomarkers could facilitate widespread clinical applications and improve personalised treatment strategies. Furthermore, integrating advanced imaging techniques and artificial intelligence could support non-invasive monitoring of lesion-level imaging biomarkers, while serial blood sampling remains necessary to measure circulating immune or tumor-derived biomarkers. Such advancements could enhance treatment efficacy by providing better insights into the interactions between immunotherapy and radiotherapy, ultimately leading to more effective cancer treatment strategies.

### 5.3 Radiation dose, fractionation and particle type

The use of radiotherapy in combination with immune checkpoint inhibitors to maximize the anti-cancer immune response remains controversial. One critical factor in addressing this issue is the need for more evidence on radiation dose effects. Over the past few decades, conventionally fractionated radiotherapy has typically employed doses of 1.8–2.0 Gy per fraction, 5 days per week for 4–8 weeks, whereas hypofractionated radiotherapy delivers higher doses in fewer fractions. This difference implies that biological responses to these two dosing regimens vary significantly. Therefore, studying molecular interactions in conventionally fractionated radiotherapy, hypofractionated radiotherapy, and their combination with immunotherapy is essential to determine the optimal dose, fractionation schedule, and treatment strategy that balances efficacy, minimal toxicity, and maximum safety.

Beyond the issue of radiation dose, the type of radiation used is another critical factor in cancer treatment. Over the past few decades, conventional photon radiation, including X-rays and γ-rays, has been widely utilized in clinical cancer therapy. However, the use of charged particles, such as carbon ions, has been rapidly increasing in recent years. In preclinical melanoma models, carbon-ion radiotherapy combined with anti-PD-1 therapy showed stronger tumor-growth suppression than conventional radio-immunotherapy. In a 4T1 mammary tumor model, carbon-ion therapy showed distinct immune modulation from photon therapy, suggesting potential relevance for future studies (Spina et al., 2021; Zhou et al., 2022). Therefore, further research is needed to explore the molecular interactions involved in heavy-ion radiotherapy combined with immunotherapy, because this will help identify new therapeutic targets and provide stronger evidence for optimizing cancer treatment outcomes.

### 5.4 Toxicity and safety of combined treatment

Combination therapy may pose greater risks due to potential adverse effects associated with both radiotherapy and immunotherapy. Radiation exposure can lead to complications such as radiation pneumonitis and dermatitis, while immune-related adverse events may occur during treatment, after prolonged exposure, or after discontinuation; risk depends on treatment class, sequence, irradiated organ and patient factors. Although numerous clinical trials have been conducted to evaluate combination strategies, toxicological assessments and protective measures must be further addressed to ensure treatment safety and minimize adverse effects.

### 5.5 Biomarkers and patient selection

With the rapid advancement of high-throughput sequencing technologies, increasing numbers of biomarkers have been identified in molecular interactions beyond those previously mentioned. For example, gut-microbiome features and microbial metabolites have been associated with cancer outcomes and responses to some therapies, but causality, assay standardization and clinical utility remain unestablished (Cho et al., 2024). Additionally, investigating biomarkers through protein post-translational modification omics during cancer therapy represents a cutting-edge research direction, offering potential druggable targets for improving cancer treatment outcomes.

A practical biomarker strategy for combined radiotherapy and immunotherapy has to answer two different questions, and most current candidates answer only one of them. The first question is whether the tumor can generate an immune response after irradiation. It is addressed by tumor-intrinsic markers, including PD-L1 expression, tumor mutational burden and mismatch repair status, and, more specifically for this setting, by the integrity of cGAS–STING pathway components, whose loss or epigenetic suppression in some tumor types may impair radiation-induced type I interferon signaling, although predictive value remains to be clinically validated. The second question is whether the patient retains an effector compartment capable of executing that response. It is addressed by host and dynamic markers, including the absolute lymphocyte count and its nadir during treatment, the estimated dose to circulating blood, the T cell receptor repertoire measured before and after irradiation, and circulating tumor DNA or exosomal double-stranded DNA as a non-invasive proxy for the interferogenic DNA released from the irradiated field. Radiomic estimates of CD8 infiltration may enable non-invasive assessment across multiple lesions when imaging quality and model domain are appropriate, but require external validation and lesion-level calibration (Huang et al., 2024).

### 5.6 Nanotechnology-enabled delivery

Radiotherapy remains a standard component of treatment for many cancers, particularly in localized or locoregionally advanced disease, and the emergence of immunotherapy has introduced new possibilities for enhancing radiotherapy outcomes. However, despite its promise, immunotherapy still carries the risk of off-target effects, even if at a lower rate. Improving immunotherapy effectiveness is complex and depends on multiple factors, including cancer antigen expression, antigen presentation, and the activation, transport, and infiltration of immune cells to the tumor site. In addition to established and investigational immunotherapy approaches, nanotechnology-based delivery platforms are being investigated as strategies to enhance antitumor immune responses. Nanomaterials are being developed to improve the delivery, pharmacokinetics or tumor localization of selected immunotherapeutic agents, although consistent clinical outcome benefits remain to be established for many platforms. However, as an emerging technology, the safety of nanomaterials in human health must be carefully considered. Some nanomaterials are difficult to degrade within the body, and long-term exposure may lead to accumulation and potential safety risks. Additionally, the molecular interactions between nanomaterials and immunotherapy require further investigation to understand their impact fully. Future nanotechnology-mediated immunotherapy should prioritise safety assessments and potential side effects to ensure its viability as a clinical treatment option.

### 5.7 From a local to a systemic view of radiotherapy

Indeed, we should shift from a “local” to a “systemic” perspective. Traditionally, radiotherapy has been seen as a local treatment method. Now, biological research reveals its powerful systemic immune regulatory effects. This requires researchers not only to focus on DNA damage in tumor cells, but also to pay attention to the changes in the entire network caused by immune cell activation, cytokine release, antigen presentation, and so on. Molecular interactions may be the material basis for all biological functions. Radiotherapy triggers a series of signaling pathways (protein–protein interactions) by causing DNA double strand breaks (disrupting protein–DNA interactions). Under some conditions, radiation-induced DNA damage and cellular stress can promote immunogenic tumor-cell death or senescence-associated inflammatory signaling, leading to release of antigens and danger-associated molecules such as ATP and HMGB1 that may enhance dendritic-cell activation and subsequent T-cell priming. Immunotherapy drugs (such as PD-1 antibodies) block the protein–protein interaction between PD-1 on the surface of T cells and PD-L1 on the surface of tumor cells, thereby relieving the inhibition of T cells and enabling them to effectively attack tumors. Basic biological research should be used to transform the synergistic phenomenon of “1 + 1>2” observed in clinical practice into predictable and optimized precision treatment plans. For example, PD-L1 expression, tumor mutational burden and immune-cell infiltration are candidate stratification variables, but none is validated across tumor types to predict the incremental benefit of adding radiotherapy to immunotherapy. Multidimensional molecular interaction research should serve as the engine that drives the optimization and innovation of this powerful combination strategy of radiotherapy and immunotherapy, ultimately bringing new hope to cancer patients.

Collectively, several critical questions must be addressed to optimize the combination of radiotherapy and immunotherapy for cancer treatment. Determining the optimal sequence and schedule for combining radiotherapy with immunomodulators is essential to maximize therapeutic efficacy. Selecting the most suitable types of immunotherapy for different cancers and understanding their effects on healthy tissues remain key challenges. Identifying reliable biomarkers to screen for the most effective immunoradiotherapy drugs could improve patient selection and treatment outcomes. Additionally, overcoming drug resistance, increasing the proportion of responsive patients, and expanding the range of immune checkpoint inhibitors are crucial for enhancing the success of combination therapy. Addressing these challenges will be fundamental in enabling more cancer patients to benefit from the synergistic effects of radiotherapy and immunotherapy.

## Data availability statement

Data sharing is not applicable to this article, as no new data were created or analysed in this study.

## Declaration of competing interest

The authors declare that they have no known competing financial interests or personal relationships that could have appeared to influence the work reported in this paper.
